# Quantifying heterologous gene expression during ectopic MazF production in *Escherichia coli*

**DOI:** 10.1186/s13104-022-06061-9

**Published:** 2022-05-13

**Authors:** Nela Nikolic, Martina Sauert, Tanino G. Albanese, Isabella Moll

**Affiliations:** 1 Institute of Science and Technology Austria (ISTA), Klosterneuburg, Austria; 2 Department of Microbiology, Immunobiology and Genetics, Max Perutz Labs, Vienna Biocenter (VBC), University of Vienna, Vienna, Austria; 3 Present Address: Living Systems Institute, University of Exeter, Exeter, UK

**Keywords:** Bacteria, Toxin–antitoxin system, *mazEF*, Flow cytometry, Heterologous gene expression, Population heterogeneity

## Abstract

**Objective:**

MazF is a sequence-specific endoribonuclease-toxin of the MazEF toxin–antitoxin system. MazF cleaves single-stranded ribonucleic acid (RNA) regions at adenine–cytosine–adenine (ACA) sequences in the bacterium *Escherichia coli*. The MazEF system has been used in various biotechnology and synthetic biology applications. In this study, we infer how ectopic *mazF* overexpression affects production of heterologous proteins. To this end, we quantified the levels of fluorescent proteins expressed in *E. coli* from reporters translated from the ACA-containing or ACA-less messenger RNAs (mRNAs). Additionally, we addressed the impact of the 5′-untranslated region of these reporter mRNAs under the same conditions by comparing expression from mRNAs that comprise (canonical mRNA) or lack this region (leaderless mRNA).

**Results:**

Flow cytometry analysis indicates that during *mazF* overexpression, fluorescent proteins are translated from the canonical as well as leaderless mRNAs. Our analysis further indicates that longer *mazF* overexpression generally increases the concentration of fluorescent proteins translated from ACA-less mRNAs, however it also substantially increases bacterial population heterogeneity. Finally, our results suggest that the strength and duration of *mazF* overexpression should be optimized for each experimental setup, to maximize the heterologous protein production and minimize the amount of phenotypic heterogeneity in bacterial populations, which is unfavorable in biotechnological processes.

**Supplementary Information:**

The online version contains supplementary material available at 10.1186/s13104-022-06061-9.

## Introduction

MazF is the toxin part of the bacterial toxin–antitoxin MazEF module, neutralized by the MazE antitoxin and thus inactive in non-stressful conditions [1, 2]. MazF acts as an endoribonuclease that cleaves single-stranded RNA regions at ACA trinucleotide sites in *Escherichia coli* [3, 4]. As artificially produced MazF degrades the rRNA precursors, as well as mRNAs [4–8], overall translation is reduced, which leads to decline in bacterial growth [9]. Even though cells grow slowly during *mazF* overexpression, cellular processes such as transcription and translation are not halted during this growth reduction [10, 11]. A previous study has shown that bacterial populations maintain transcription during MazF production, possibly to ensure synthesis of important components of translational machinery and the antitoxin MazE, so the cells could recover from the stress rapidly [11]. Moreover, during *mazF* overexpression protein synthesis is possible from mRNAs that do not contain ACA sequences, and thus are not targeted by MazF [10]. Ectopic MazF production has been included in various experimental frameworks in biotechnology and synthetic biology. For instance, *mazF* overexpression has been employed to improve high-yield production of the protein of interest [12, 13], for manipulation of cellular resources [14], and in examining interaction networks within synthetic microbial communities [15]. In this study, we investigate how basic mRNA properties, such as the mRNA sequence and length of the untranslated region, affect synthesis of the heterologous protein during *mazF* overexpression. Additionally, this study underlines the importance of addressing the connection between expression of the gene of interest and phenotypic heterogeneity in bacterial populations during *mazF* overexpression.

## Main text

### Methods

#### Bacterial strains and reporter systems

We employed three constitutively expressed reporter systems: a plasmid-based *gfp* reporter gene devoid of ACA sites (*gfp*_ΔACA_), transcribed into (1) a canonical or (2) a leaderless mRNA [16], and (3) a chromosomally encoded *mCherry* reporter gene with its native ACA sites [9]. The coding *gfp*_ΔACA_ mRNA region is thus not targeted by MazF [16], while the *mCherry* mRNA is prone to the MazF-mediated cleavage [9]. Low- or high-copy plasmids harboring *gfp*_ΔACA_ reporter systems were transformed into strain TB212, which is a derivative of *E. coli* BW27784 that constitutively transports l-arabinose (Ara) without metabolizing it [17]. Strain TB212 carries a chromosomally integrated *mCherry* reporter gene placed under the phage λ promoter [18], and it is additionally transformed with plasmid pBAD-*mazF* [19]. All *gfp*_ΔACA_ reporter systems [16, 20], strains [17, 21] and plasmids are listed in Additional file 1: Table S1. Bacterial growth was monitored by measuring optical density at 600 nm (OD_600_), and flow cytometry analysis was performed with LSR Fortessa (BD, USA). Detailed experimental protocols, flow cytometry setups and analyses are described in [9].

#### Fluorescence analysis

As a negative control for GFP and mCherry fluorescence, we measured autofluorescence of strain BW27784 pBAD-*mazF*. As an additional negative GFP fluorescence control, we employed strain TB212 pBAD-*mazF* with a *gfp*_ΔACA_ reporter gene system in which a stem loop structure was placed closely upstream of the start codon to prevent ribosome binding and consequently translation, located on a high-copy or a low-copy plasmid (Additional file 2). Normalized GFP and mCherry fluorescence was calculated as the mean fluorescence level of a TB212 pBAD-*mazF gfp*_ΔACA_ reporter strain divided by the mean fluorescence level of the reporterless strain BW27784 pBAD-*mazF*, measured at the same time point. The percentage increase in fluorescence between two measurements, e.g. between two time points t, was calculated on normalized fluorescence values as increase = [(fluorescence(t_2_) − fluorescence(t_1_))/fluorescence(t_1_)] * 100. Error bars in all graphs present standard deviation. To evaluate differences in fluorescence datasets we used two-tailed, paired Student’s *t*-test (induced vs. uninduced cultures, or 6 h-induction vs. 2 h-induction).

#### Sequence analysis

A 910 nucleotide-region comprising the *mazEF* locus was analyzed in the strains K-12 MG1655 (NCBI ID: U00096.3, range: 2,910,556–2,911,465), K-12 BW25113 (NCBI ID: CP009273.1, range: 2,903,915–2,904,824), and BL21(DE3) (NCBI ID: CP053602.1, range: 2,744,443–2,745,352).

### Results and discussion

#### Low-level translation of the leaderless *gfp* mRNA throughout bacterial growth phases

**Fig. 1:**
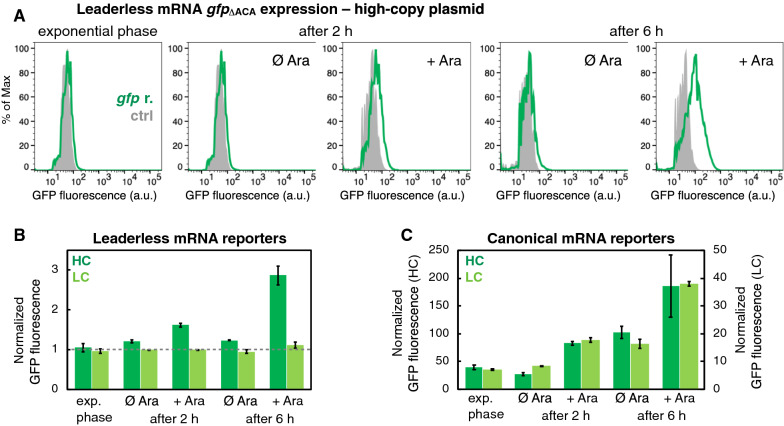
Flow cytometry analysis of GFP fluorescence encoded by *gfp*_ΔACA_ reporters. The leaderless mRNA of the ll-*gfp*_ΔACA_ reporter entirely lacks a 5′-UTR, and this reporter construct has the start sequence ATG of the *gfp*_ΔACA_ gene following directly after the promoter region [16, 20]. The canonical mRNA of the can-*gfp*_ΔACA_ reporter comprises a 5′-UTR, which includes a strong ribosome binding site. **A** Green distributions depict measurements of the *E. coli* strain TB212 harboring the plasmid pBAD-*mazF* and the ll-*gfp*_ΔACA_ reporter encoded on a high-copy plasmid. Light grey distributions depict measurements of the strain harboring only the plasmid pBAD-*mazF*. Here, one replicate is presented, for further results see Additional file 2. Ectopic *mazF* overexpression from plasmid pBAD-*mazF* [19] was induced by adding 0.1% Ara in the early exponential phase, at OD_600_ = 0.18–0.22. Flow cytometry analysis was performed in the early exponential phase, and 2 h [average OD_600_(uninduced) = 2.45, OD_600_(*mazF*-induced) = 0.45] and 6 h after *mazF* overexpression [average OD_600_(uninduced) = 4.42, OD_600_(*mazF*-induced) = 0.80]. **B** Normalized GFP fluorescence from the ll-*gfp*_ΔACA_ reporters or **C** can-*gfp*_ΔACA_ reporters encoded on a high-copy (HC, dark green) or a low-copy (LC, light green) plasmid, measured in different phases of bacterial growth and after adding arabinose (Ara) to induce *mazF* expression (N = 3 independent replicate cultures). Altogether, the growth of *mazF*-induced cultures was reduced by 77–86% after 2 h, and by 71–90% after 6 h, compared to the respective uninduced controls, see Additional file 2

In this study, we sought to determine how the length of the untranslated region (UTR) of an mRNA affects the synthesis of the corresponding protein throughout bacterial growth phases, and specifically during *mazF* overexpression. Bacterial canonical mRNAs harbor a 5′-UTR comprising ribosome recognition regions and other translational signals, as reviewed in [22]. Leaderless mRNAs lack 5′-UTRs or possess very short 5′-UTRs, and are, in general, translated less efficiently than canonical mRNAs. Nonetheless, previous in vitro and in vivo studies have shown that *E. coli* leaderless mRNAs can be translated by different ribosome variants [23–25]. In our experiments, we analyzed the GFP fluorescence as a proxy for translation of the leaderless *gfp*_ΔACA_ reporter, referred to as ll-*gfp*_ΔACA_ reporter, and the canonical mRNA *gfp*_ΔACA_ reporter, referred to as can-*gfp*_ΔACA_ reporter. Analysis of the ll-*gfp*_ΔACA_ reporters in the early exponential phase showed that GFP fluorescence was not significantly higher in populations harboring the fluorescent reporter compared to control populations that did not carry the fluorescent reporter (Fig. 1A, B). This indicates very low ll-*gfp*_ΔACA_ expression in the early exponential phase, in contrast to the higher levels of expression of the can-*gfp*_ΔACA_ reporters (Fig. 1C). However, we detected an increased fluorescent signal as a result of translation of the ll-*gfp*_ΔACA_ mRNA in the later phases of bacterial growth, as well as 2 and 6 h after inducing *mazF* expression (Table 1, Part A). After 2 h, GFP fluorescence of *mazF*-induced cultures increased on average by 34% compared to the respective uninduced cultures, when the ll-*gfp*_ΔACA_ reporter was encoded on a high-copy plasmid (see “Methods” for the calculation). Six hours after *mazF* overexpression, GFP fluorescence of *mazF*-induced cultures increased by 133%. These results were further corroborated with the biochemical analysis (Additional file 3: Fig. S1). When the ll-*gfp*_ΔACA_ reporter was encoded on a low-copy plasmid, GFP fluorescence of *mazF*-induced cultures did not significantly change 2 h after *mazF* overexpression, however after 6 h GFP fluorescence of *mazF*-induced cultures increased by 17%. In parallel, we analyzed GFP fluorescence encoded by the can-*gfp*_ΔACA_ reporters (Fig. 1C, Table 1, Part A). Six hours after *mazF* overexpression, GFP fluorescence of *mazF*-induced cultures increased on average by 79% when the can-*gfp*_ΔACA_ reporter was encoded on a high-copy plasmid (one replicate culture did not yield a significant GFP fluorescence increase), and by 134% when the can-*gfp*_ΔACA_ reporter was encoded on a low-copy plasmid. In all cases, the increase in GFP fluorescence indicates the fluorescent protein synthesis and its accumulation inside bacterial cells during *mazF* overexpression. Together, this analysis suggests that translation of leaderless mRNAs occurs throughout the bacterial growth phases as well as during *mazF* overexpression, albeit at low levels.

**Table 1:** Summary table: Fluorescence increase analysis

A. Fluorescence increase of *mazF*-induced cultures compared to the uninduced cultures	% increase	p-value
Leaderless *gfp*_ΔACA_ reporter		
High-copy plasmid, after 2 h	34.4 ± 2.9^a^	0.002
High-copy plasmid, after 6 h	132.8 ± 17.9	0.006
Low-copy plasmid, after 2 h	− 0.1 ± 0.6	ns^b^
Low-copy plasmid, after 6 h	17.1 ± 4.0	0.021
Canonical *gfp*_ΔACA_ reporter		
High-copy plasmid, after 2 h	203.0 ± 19.0	0.0003
High-copy plasmid, after 6 h	79.4 ± 39.2	ns
Low-copy plasmid, after 2 h	112.7 ± 14.0	0.004
Low-copy plasmid, after 6 h	133.9 ± 20.2	0.001

B. Fluorescence increase during *mazF* overexpression: 6 h after induction compared to 2 h after induction	% increase	p-value
Leaderless *gfp*_ΔACA_ reporter		
High-copy plasmid	77.6 ± 13.4	0.01
Low-copy plasmid	12.3 ± 6.6	ns
Canonical *gfp*_ΔACA_ reporter		
High-copy plasmid	123.9 ± 60.0	ns
Low-copy plasmid	113.4 ± 5.6	1.3E−05
*mCherry* reporter		
Chromosomal	34.7 ± 27.9	0.001

^a^mean ± standard deviation

^b^ns stands for not significant

#### Increased cellular concentration of GFP proteins translated from ACA-less mRNAs during *mazF* overexpression

**Fig. 2:**
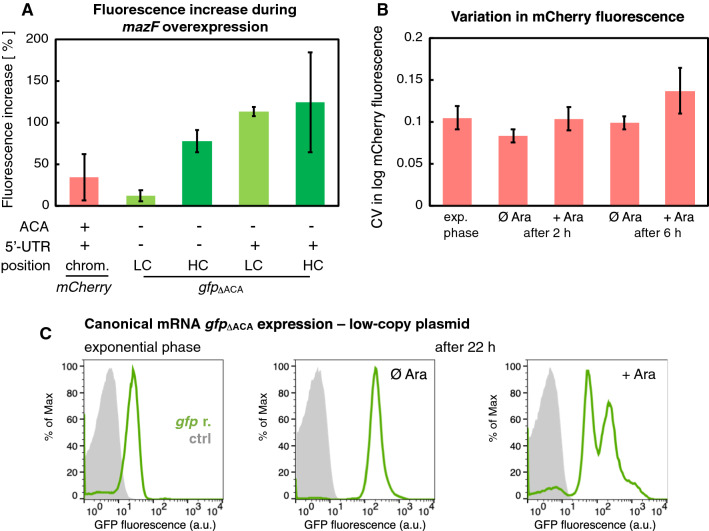
Increase in the mean level and variation in fluorescence during *mazF* overexpression. **A** The increase in fluorescence of bacterial cultures was determined by comparing two flow cytometry time points, measured 2 and 6 h after inducing *mazF* expression. The fluorescent gene reporters were encoded on a high-copy (HC) or a low-copy plasmid (LC), transcribed to a leaderless (without 5′-UTR) or a canonical (containing 5′-UTR) mRNA that contained ACA sites (*mCherry*) or was devoid of ACA sites (*gfp*_ΔACA_). The highest increase in fluorescence was detected from the can-*gfp*_ΔACA_ reporter encoded on a high-copy plasmid, which was almost twofold higher than the fluorescence increase measured from the ll-*gfp*_ΔACA_ reporter encoded on a high-copy plasmid (N = 3 independent replicate cultures for GFP fluorescence analysis, N = 12 for mCherry fluorescence analysis). **B** Coefficient of variation (CV) in mCherry fluorescence was calculated as standard deviation divided by the mean of the log_10_-transformed fluorescence data, for different phases of bacterial growth, and it is a proxy for population heterogeneity [9]. CV in mCherry fluorescence increased by 32.4 ± 19.4% in *mazF*-induced cultures, during 4 h of *mazF* overexpression (N = 12 independent replicate cultures, p-value = 0.0001). **C** Green distributions depict measurements of the *E. coli* strain BW27784 harboring the plasmid pBAD-*mazF* and the can-*gfp*_ΔACA_ reporter encoded on a low-copy plasmid. Light grey distributions depict measurements of the strain harboring only the plasmid pBAD-*mazF*. 0.02% Ara was added to exponentially growing cultures to induce *mazF* overexpression, and flow cytometry analysis was performed in the early exponential phase, and 22 h after *mazF* overexpression [OD_600_(uninduced) = 5.31, OD_600_(*mazF*-induced) = 3.20]. After 22 h, *mazF*-induced cultures were comprised of bacterial subpopulations of different GFP fluorescence intensities, while uninduced cultures exhibited unimodal distributions of GFP fluorescence

We analyzed differences in fluorescence intensity of bacterial cells between two measurements during *mazF* overexpression, i.e. 2 and 6 h after inducing *mazF* expression (Fig. 2A, Table 1, Part B). We measured the fluorescence from reporter proteins encoded by the ACA-containing *mCherry*, the can-*gfp*_ΔACA_ reporter genes transcribed into canonical mRNAs, as well as the ll-*gfp*_ΔACA_ reporter gene transcribed into a leaderless mRNA. Our analysis shows that mCherry fluorescence increased only slightly during 4 h of *mazF* overexpression, by 35% on average (red column in Fig. 2A). In the same experimental setup, GFP fluorescence from the ll-*gfp*_ΔACA_ reporter increased by 78% when the reporter was encoded on a high-copy plasmid, and by 12% when encoded on a low-copy plasmid. The highest fluorescence increase was measured for cells carrying the can-*gfp*_ΔACA_ reporter: GFP fluorescence increased by 124% when the reporter was encoded on a high-copy plasmid, and by 113% when encoded on a low-copy plasmid. Firstly, the overall fluorescence increase corroborates previous findings that transcription and translation carry on during *mazF* overexpression [10, 11]. The growth rate reduction during *mazF* overexpression also indirectly contributes to the increased level of fluorescence because highly stable reporter proteins, such as GFP and mCherry, are less diluted through slower cell division [9]. Secondly, a higher fluorescence increase for cells harboring *gfp*_ΔACA_ reporters indicates a higher rate of protein synthesis from mRNAs devoid of ACA sites than from ACA-containing mRNAs that can be cleaved by MazF, such as the *mCherry* mRNA, which was already implied in [10]. Finally, these results suggest considerably higher expression of proteins translated from the canonical mRNA than the leaderless mRNA form during ectopic *mazF* expression.

#### Interplay between gene expression and population heterogeneity during *mazF* overexpression

A previous study has established that fluorescence encoded by the reporter gene placed under the phage λ promoter P_R_, can be employed to quantify population heterogeneity during *mazF* overexpression [9]. Specifically, differences in the reporter protein fluorescence reflect changes in the single-cell growth rate, indicating that increased variation in the growth rates of single cells within the population underlies increased population heterogeneity. In this study, population heterogeneity measured as the variation in mCherry fluorescence increased by 32% during 4 h of *mazF* overexpression (Fig. 2B). In addition, it has been previously shown that the formation of bacterial subpopulations of different fluorescent intensities occurs 5.5–6.5 h after inducing *mazF* overexpression, regardless of whether the constitutively expressed fluorescent gene reporters are encoded in the chromosome or on a plasmid [9]. Our analysis indicates that bacterial subpopulations exhibited distinct levels of can-*gfp*_ΔACA_ expression 22 h after inducing *mazF* overexpression (Fig. 2C). Longer *mazF* overexpression likewise promoted larger differences in the fluorescence measurements between the replicate cultures (see error bars in Figs. 1C and 2A), and in one case even resulted in an insignificant fluorescence increase in can-*gfp*_ΔACA_ expression (Table 1), possibly due to pleiotropic effects of *mazF* overexpression. To conclude, even though prolonged *mazF* overexpression commonly increases GFP fluorescence encoded by both ll-*gfp*_ΔACA_ and can-*gfp*_ΔACA_ reporters measured at the population level, it also promotes bacterial population heterogeneity.

In general, *E. coli* strains overexpressing an ACA-less gene have been used for commercial production of the respective protein under optimized fermentation conditions and *mazF* overexpression [12, 13, 26]. Recombinant protein production during *mazF* overexpression can be maintained for 3 [10], 4 [13] or even 7 days [12]. However, ectopic MazF production non-uniformly alters growth rates of bacterial cells [9]. Bacterial population heterogeneity is typically unfavorable in biotechnological processes as it reduces the yield of recombinant protein production, and causes process instability especially during large-scale cultivation [27]. Inducing *mazF* expression at lower levels or shorter periods of time has a lesser impact on the population heterogeneity [9, 28, 29]. Furthermore, one of the most widely used hosts for recombinant protein production, *E. coli* BL21(DE3) [10, 12, 13], contains *mazEF* locus 100% identical to the *mazEF* locus of the here studied K-12 strain (see “Methods”). Therefore, a genetically engineered host *E. coli* strain with deleted *mazEF* locus could be employed in biotechnological setups, as the presence of the native *mazEF* locus has been shown to be the main source of population heterogeneity during ectopic *mazF* overexpression [9].

### Conclusions

Our results show that *mazF* overexpression considerably increases cellular concentration of fluorescent proteins translated from mRNAs devoid of ACA sites. The higher reporter protein fluorescence is observed when the reporter gene is expressed at higher levels, which can be achieved by (1) employing a reporter system with a strong promoter and a strong ribosome binding site, and (2) inserting the reporter system on a high-copy plasmid. This suggests that genetic systems with different transcriptional and translational properties can be used to study cellular resource allocation during *mazF* overexpression [14]. Moreover, current efforts in synthetic biology and biotechnology focus on engineering bacterial systems with reduced phenotypic population heterogeneity [30, 31]. In order to avoid increased population heterogeneity emerging during ectopic MazF production, and to maintain the stability of recombinant protein synthesis, it is necessary to optimize experimental setups that employ MazF by adjusting the strength and duration of *mazF* overexpression.

## Limitations

This study would benefit from further analysis of different types of fluorescent reporter systems in different *E. coli* strains, to provide a better understanding of the limits of experimental frameworks when employing *mazF* overexpression for the production of the specific protein and manipulation of synthetic circuits.

## Supplementary Information

**Additional file 1: Table S1.** List of strains and plasmids.

**Additional file 2.** Flow cytometry data for figures.

**Additional file 3: Figure S1.** Analysis of the leaderless reporter. Additional methods.

## Data Availability

The datasets supporting the conclusions of this article are included within the article and its Additional file 2. Flow cytometry data supporting the conclusions of this article are available in the FlowRepository (http://flowrepository.org) with assigned Repository IDs: FR-FCM-Z3UV (reporter fluorescence data), FR-FCM-Z4MB (Fig. 2C) and FR-FCM-Z3VY (additional negative controls for GFP fluorescence).

## Abbreviations

RNA: Ribonucleic acid
mRNA: Messenger RNA
ACA sequence: Adenine–cytosine–adenine sequence
GFP: Green fluorescent protein
*gfp*_ΔACA_: *gfp* Reporter gene devoid of ACA sites
ll-*gfp* reporter: Leaderless *gfp* reporter
can-*gfp* reporter: Canonical *gfp* reporter
OD_600_: Optical density at 600 nm
Ara: Arabinose
