# Correction to: Quantifying heterologous gene expression during ectopic MazF production in *Escherichia coli*

**DOI:** 10.1186/s13104-022-06152-7

**Published:** 2022-07-25

**Authors:** Nela Nikolic, Martina Sauert, Tanino G. Albanese, Isabella Moll

**Affiliations:** 1 Institute of Science and Technology Austria (ISTA), Klosterneuburg, Austria; 2 Department of Microbiology, Immunobiology and Genetics, Max Perutz Labs, Vienna Biocenter (VBC), University of Vienna, Vienna, Austria; 3 Present Address: Living Systems Institute, University of Exeter, Exeter, UK

## Correction to: BMC Research Notes (2022) 15:173 https://doi.org/10.1186/s13104-022-06061-9

Following the publication of the original article [1], the second panel of Fig. 1A was amended, and now shows the correct GFP fluorescence histogram (time point: after 2 h, condition: Ø Ara).

**Fig. 1:**
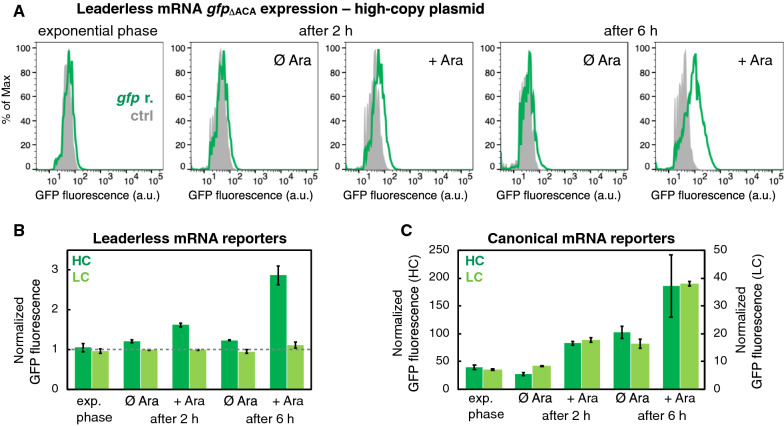
Flow cytometry analysis of GFP fluorescence encoded by *gfp*_ΔACA_ reporters. The leaderless mRNA of the ll-*gfp*_ΔACA_ reporter entirely lacks a 5′-UTR, and this reporter construct has the start sequence ATG of the *gfp*_ΔACA_ gene following directly after the promoter region [16, 20]. The canonical mRNA of the can-*gfp*_ΔACA_ reporter comprises a 5′-UTR, which includes a strong ribosome binding site. **A** Green distributions depict measurements of the *E. coli* strain TB212 harboring the plasmid pBAD-*mazF* and the ll-*gfp*_ΔACA_ reporter encoded on a high-copy plasmid. Light grey distributions depict measurements of the strain harboring only the plasmid pBAD-*mazF*. Here, one replicate is presented, for further results see Additional file 2. Ectopic *mazF* overexpression from plasmid pBAD-*mazF* [19] was induced by adding 0.1% Ara in the early exponential phase, at OD_600_ = 0.18–0.22. Flow cytometry analysis was performed in the early exponential phase, and 2 h [average OD_600_(uninduced) = 2.45, OD_600_(*mazF*-induced) = 0.45] and 6 h after *mazF* overexpression [average OD_600_(uninduced) = 4.42, OD_600_(*mazF*-induced) = 0.80]. **B** Normalized GFP fluorescence from the ll-*gfp*_ΔACA_ reporters or **C** can-*gfp*_ΔACA_ reporters encoded on a high-copy (HC, dark green) or a low-copy (LC, light green) plasmid, measured in different phases of bacterial growth and after adding arabinose (Ara) to induce *mazF* expression (N = 3 independent replicate cultures). Altogether, the growth of *mazF*-induced cultures was reduced by 77–86% after 2 h, and by 71–90% after 6 h, compared to the respective uninduced controls, see Additional file 2

Figure 1A has been solely used to visualize the flow cytometry data, thus the Figure legend remains unchanged.

This correction does not affect any analysis included in the published article, the reported results, or the interpretation of the results.

The original article has been corrected.
