# Supplementary material for: Quantifying heterologous gene expression during ectopic MazF production in Escherichia coli
Source: BMC Res Notes. 2022 May 13;15:173. doi: 10.1186/s13104-022-06061-9 (PMC9102682; doi:10.1186/s13104-022-06061-9)
Supplement: Supplementary file 1 — Additional file 1: Table S1. List of strains and plasmids. [file 13104_2022_6061_MOESM1_ESM.pdf]

Table S1 List of strains and plasmids

Strain or plasmid name	Relevant characteristics	Source or reference
TOP10	Strain for plasmid propagation	Moll Lab Collection, *#497
BW27784	K-12 strain derivative (ancestor strain: BW25113) used for arabinose-inducible expression; Δ(*araD-araB*)567, Δ*lacZ*4787(::*rrnB*-3), *λ*-, Δ(*araH-araF*)570(::frt), Δ*araE*p-532::frt, φPcp18*araE*533, Δ(*rhaD-rhaB*)568, *hsdR*514<br>Reporterless strain	[17], #1056
TB212	BW27784 attP21::λP_R-*mCherry*::frt; constitutive *mCherry* expression	[18], #1058
MG1655	Wild-type *E. coli* K-12 F-*, λ-, ilvG-, rfb-50, rph-1*	[21], #103
pUH-C-leaderlessΔACA-EmGFP (pMS2_53)	AmpR, pUH-C; leaderless Em*gfp*ΔACA expression	[16, 20], #863
pUH-C-canonical-EmGFP (pMS2_612)	AmpR, pUH-C w/o 2nd operator; constitutive Em*gfp*ΔACA expression	[16, 20], #884
pUH-C-ΔTIR-ΔACA-EmGFP (pMS2_645)	AmpR, pUH-C w/o 2nd operator; stem loop structure upstream of Em*gfp*ΔACA; negative control for the fluorescence readout	[16, 20], #886
pZS*12-GFP	AmpR, SC101* ori	Guet Lab Collection, #1059
pZS*-53	AmpR, SC101* ori, HindIII/XhoI digestion of the insert from pMS2_53 to pZS*12-GFP; leaderless Em*gfp*ΔACA expression	This study, #1060
pZS*-612	AmpR, SC101* ori, HindIII/XhoI digestion of insert from pMS2_612 to pZS*12-GFP; constitutive Em*gfp*ΔACA expression	[9], #1061
pZS*-645	AmpR, SC101* ori, HindIII/XhoI digestion of the insert from pMS2_645 to pZS*12-GFP; control for fluorescence readout	This study, #1063
pBAD-*mazF*	CamR, p15A ori, pBAD33 backbone, P_{BAD}-*mazF*; Ara-inducible *mazF* expression	[19], #692

* # is the position in the Moll Lab Collection
