# Supplementary material for: Quantifying heterologous gene expression during ectopic MazF production in Escherichia coli
Source: BMC Res Notes. 2022 May 13;15:173. doi: 10.1186/s13104-022-06061-9 (PMC9102682; doi:10.1186/s13104-022-06061-9)
Supplement: Supplementary file 3 — Additional file 3: Figure S1. Analysis of the leaderless reporter. Additional methods. [file 13104_2022_6061_MOESM3_ESM.pdf]

Additional Figure and Methods

Quantifying heterologous gene expression during ectopic MazF production in *Escherichia coli*

By

Nela Nikolic, Martina Sauert, Tanino G. Albanese, and Isabella Moll

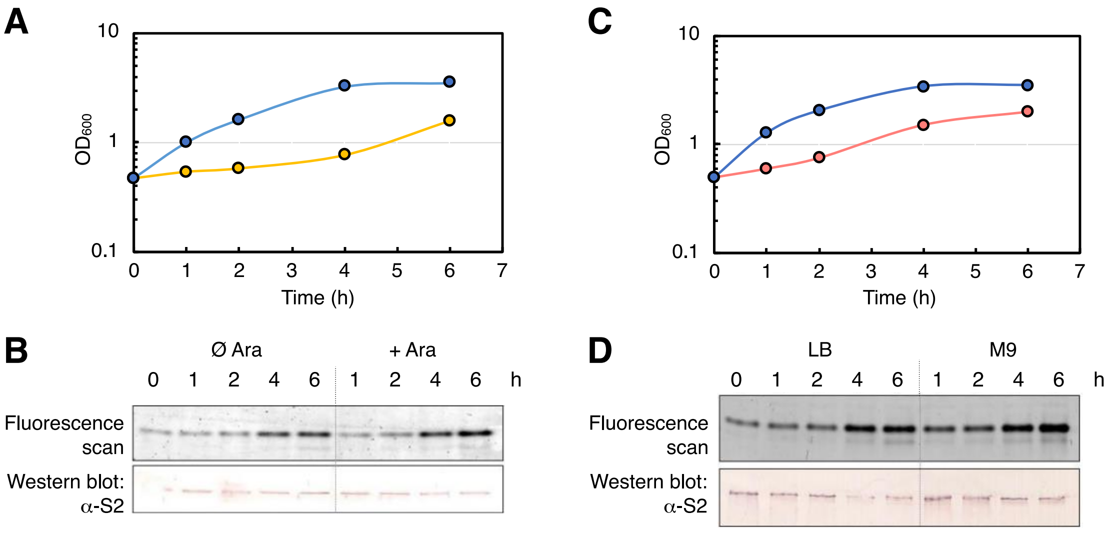

Additional Figure S1. Analysis of the leaderless reporter ll-*gfp*$_{\Delta ACA}$

A) The *E. coli* strain MG1655 pBAD-*mazF* harboring a high-copy plasmid with the ll-*gfp*$_{\Delta ACA}$ reporter was cultivated in LB rich medium. At OD_{600} of ~0.5, the culture was separated in two flasks, and 0.5% Ara was added to one flask to induce *mazF* overexpression (yellow) while the other flask served as the uninduced control (blue). Bacterial growth was monitored by measuring OD_{600} at indicated time points. **B)** Cell lysates from samples taken from **A)** were resolved on native PAA gels; GFP was detected directly through fluorescence scanning before proceeding with western blotting and hybridization with α-S2 antibodies. **C)** The same strain was cultured until mid-exponential phase (OD_{600} of ~0.5) in LB rich medium (blue), and one part of the culture was washed and resuspended in M9 minimal medium supplemented with 0.4% glucose (red). **D)** Cell lysates from samples taken from **C)** and analyzed as described in **B)**. Altogether, these results indicate an increase in the amount of GFP encoded by ll-*gfp*$_{\Delta ACA}$ in the later phases of bacterial growth (time points 4 h and 6 h), in both LB rich and M9 minimal media, as well as during *mazF* overexpression.

Additional Methods for protein analysis (Figure S1B and S1D)

Native cell disruption for native PAGE. Melted cell pellets were resuspended in appropriate volumes of 1 mg lysozyme/ml 1xTE buffer resulting in 10 OD/ml (lysozyme from chicken egg white, Fluka, in a final concentration of 0.2 mg/OD), and incubated on ice for 10 min. The mixture was shock frozen in $N_{2\,aq}$ and thawed at room temperature three times. The cell debris was pelleted by centrifugation at 4°C and 30,000 rpm for 10 min, and the supernatant was used for native PAGE.

Native PAGE. Native cell lysates were mixed in a 1:1 ratio with 2x native sample buffer and immediately loaded onto native PAA gels. Electrophoresis was performed in 1x native running buffer at 30 mA per gel in Biorad Protean II electrophoresis cells. For detection of GFP signals (fluorescence scan) the gels were scanned using a Typhoon^{TM} FLA 9000 (GE Healthcare) scanner.

Western blot. Proteins separated on native PAA gels were transferred to a nitrocellulose membrane (Amersham Protan 0.2 µm) by semi-dry transfer in a Biorad Trans-Blo Semi-Dry Transfer Cell. Membranes were blocked by incubation in blocking buffer for 1 h at room temperature or overnight at 4°C. The primary antibody (α-$S2_{\alpha 2}$ from rabbit, dilution 1:5,000 in blocking buffer) was applied and incubated for 1 h at room temperature or overnight at 4°C. After 3x 10 min washes in 1xPBS-T, infrared-coupled secondary antibodies (α-Rab 1:15,000 dilution in blocking buffer) were applied for 45 min at room temperature then washed again as before. Detection on Odyssey scanner (Licor).
